# The Oldest Homœopathic Physician

**Published:** 1892-03

**Authors:** L. B. Wells

**Affiliations:** Utica, N. Y.


### THE OLDEST HOMŒOPATHIC PHYSICIAN.

EDITOR OF THE HOMŒOPATHIC PHYSICIAN:

The question who is the oldest homœopathic physician now in practice in the United States is being discussed.*

Dr. S. Seward, of Syracuse, N. Y., became a convert to Homœopathy early in the year 1847. He is a true Hahnemannian. He was eighty-one years old last September, just three weeks older than myself, L. B. WELLS,

UTICA, N. Y.

---

* See Dr. Hoopes' letter, January number, page 31.

